# Roles of Close Homologues SigB and SigD in Heat and High Light Acclimation of the Cyanobacterium *Synechocystis* sp. PCC 6803

**DOI:** 10.3390/life12020162

**Published:** 2022-01-21

**Authors:** Otso Turunen, Satu Koskinen, Juha Kurkela, Outi Karhuvaara, Kaisa Hakkila, Taina Tyystjärvi

**Affiliations:** Molecular Plant Biology, Department of Life Technologies, University of Turku, FI-20014 Turku, Finland; otso.h.turunen@utu.fi (O.T.); satkos@hotmail.com (S.K.); jhmkur@utu.fi (J.K.); outkar@utu.fi (O.K.); kaihak@utu.fi (K.H.)

**Keywords:** regulation of transcription, sigma factors, SigB, SigD, high light, heat stress

## Abstract

Acclimation of cyanobacterium *Synechocystis* sp. PCC6803 to suboptimal conditions is largely dependent on adjustments of gene expression, which is highly controlled by the σ factor subunits of RNA polymerase (RNAP). The SigB and SigD σ factors are close homologues. Here we show that the *sigB* and *sigD* genes are both induced in high light and heat stresses. Comparison of transcriptomes of the control strain (CS), ΔsigB, ΔsigD, ΔsigBCE (containing SigD as the only functional group 2 σ factor), and ΔsigCDE (SigB as the only functional group 2 σ factor) strains in standard, high light, and high temperature conditions revealed that the SigB and SigD factors regulate different sets of genes and SigB and SigD regulons are highly dependent on stress conditions. The SigB regulon is bigger than the SigD regulon at high temperature, whereas, in high light, the SigD regulon is bigger than the SigB regulon. Furthermore, our results show that favoring the SigB or SigD factor by deleting other group 2 σ factors does not lead to superior acclimation to high light or high temperature, indicating that all group 2 σ factors play roles in the acclimation processes.

## 1. Introduction

Efficient recycling and sustainable production of valuable compounds are major challenges of the future. Development of carbon neutral production systems and improvement of recycling could help to prevent further global warming and reduce pollution. Municipal, piggery, and dairy wastewaters, as well as anaerobic digestion reject waters, are typically nutrient rich, containing plenty of ammonium, nitrate, and phosphate [1,2]. Cyanobacteria are efficient in collecting nutrients [3] and many valuable products, including ethanol, isopropanol, butanol, biohydrogen and alkanes, have already been produced in cyanobacteria [4]. In the most tempting scenario, cyanobacteria are engineered to produce high value compounds, not only biomass, and simultaneously remediate wastewaters. For biotechnical applications, robust strains with high acclimation capacity are beneficial. Keeping up constant optimal environmental conditions, such as optimal temperature and constant light, is expensive, and using wastewater as a growth medium might cause additional problems; wastewaters typically contain harmful chemicals, and their nutrient balance is variable and not optimal.

Acclimation of cyanobacteria to suboptimal conditions is strongly dependent on changes in gene expression. In cyanobacteria, regulatory σ subunits of the RNA polymerase (RNAP) play major roles in acclimation responses. In optimal growth conditions, the RNAP core mainly recruits the primary σ factor that guides RNAP to transcribe housekeeping genes [5]. In suboptimal conditions, RNAP holoenzymes bearing stress-condition-specific alternative σ factor(s) become abundant and change the transcriptome to allow acclimation to that particular stress [5,6]. The cyanobacterium *Synechocystis* sp. PCC 6803 contains nine different σ factors. SigA is a primary σ factor that is mainly responsible for transcription of housekeeping genes during exponential growth; SigB, SigC, SigD, and SigE are group 2 σ factors that play major roles when cells acclimate to suboptimal conditions, and SigF, SigG, SigH, and SigI are group 3 σ factors [6]. Construction of robust cell factory strains might be possible via modification of the σ factor content of cells [7], and highly similar group 2 σ factors SigB and SigD [8,9] are of particular interest in the construction of robust production strains.

The expression of the SigB gene is typically transiently up-regulated when *Synechocystis* cells are transferred from standard conditions to stress conditions, such as high temperature [10,11], high salt [8,12,13], or from darkness to light [14]. The expression of the *sigD* gene is enhanced in high light [9,14], and cells do not acclimate properly to high light without the SigD factor [8,15].

The amounts of SigB and SigD proteins are highly dependent on environmental conditions and so is the formation of the transcription initiation competent RNAP-SigB and RNAP-SigD holenzymes. The amount of the RNAP-SigD holoenzyme decreases in darkness and increases in high light, compared to the standard conditions, whereas formation of the RNAP-SigB holoenzyme is induced by high temperature or salt treatments [5]. Furthermore, inactivation of the other group 2 σ factors influences the amount of the remaining group 2 σ factor, and the amounts of SigB and SigD proteins increase two-fold if the other group 2 σ factors are knocked out [16].

To get a more comprehensive picture of the roles of SigB and SigD factors, the performance of ΔsigB, ΔsigD, ΔsigCDE, and ΔsigBCE strains were studied in heat and high light conditions. In addition, the transcriptomes of the ΔsigB, ΔsigD, ΔsigCDE, and ΔsigBCE strains were compared in standard growth conditions and after heat and high light treatments, in order to characterize the specific regulons of SigB and SigD in these stress conditions.

## 2. Material and Methods

### 2.1. Strains 

The glucose tolerant strain of *Synechocystis* sp. PCC 6803 (CS) was used as a control and host strain [8]. The construction of ΔsigB, ΔsigD [8], ΔsigBCE, and ΔsigCDE [17] strains has been described earlier. Single and triple inactivation strains were maintained on BG-11 plates in standard growth conditions in the presence of appropriate antibiotics, as described earlier [8,17], but antibiotics were omitted from liquid cultures.

**Figure 1:**
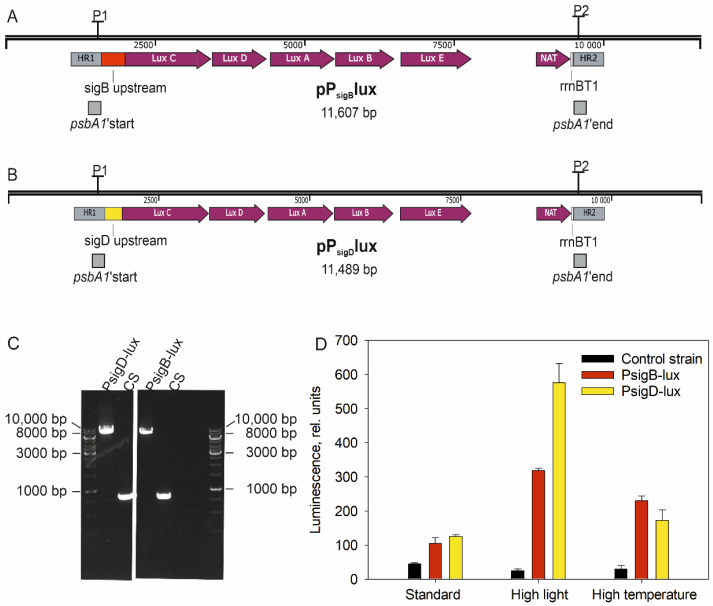
Activities of *sigB* and *sigD* promoters in high light and temperature. The 402 bp fragment upstream of the *sigB* coding region (**A**) or 284 bp fragment upstream of the *sigD* coding region (**B**) was inserted upstream of the promoter-less lux operon, and nourseotricine antibiotic cassette (NAT) was added after the lux operon for antibiotic selection. The promoter-lux-NAT construct was inserted in the middle of the non-essential *psbA1* gene, and the CS strain of *Synechocystis* was transformed with linearized plasmids to produce PsigB- and PsigD-lux strains. HR1 and HR2 are proximal areas of the *psbA1* gene used for homologous recombination. (**C**) PCR verification of PsigB- and PsigD-lux strains. The amplicon in the CS strain is 829, 8043 in PsigB-lux, and 7925 bp in PsigD-lux. The primers are marked as P1 and P2 in (**A**,**B**). (**D**) Luciferase activity was measured from control (CS), PsigB-, and PsigD-lux strains (3-day old cultures) at standard growth conditions or after 15 min of high light or temperature treatments. The luciferase substrate, 0.1 mM decanal, was added to the samples 15 min prior to measurements.

To study the *sigB* and *sigD* promoters, P_sigB_- and P_sigD_-lux strains were constructed. First, a nourseotricine resistance cassette (NAT) with the *rrnBT1* transcription terminator sequence of *E. coli* was released as a HindII fragment from plasmid pGEM-NATter (generous gift from L. Lopez-Maury), and the single stranded ends of the DNA fragment were filled with Klenow enzyme. This NAT fragment was then cloned into the SmaI site after the *luxCDABE* operon into the pTETlux plasmid (generous gift from Marko Virta) to obtain the pTETluxNAT plasmid. Either the 402 bp long fragments of upstream from the coding region of the *sigB* gene (Figure 1A) or 284 bp long upstream fragment of the *sigD* gene (Figure 1B) was used to control the expression of the *lux* operon. For insertion to the *Synechocystis* chromosome, the 200 bp long upstream and downstream regions of the *psbA1* gene were inserted to the both ends of the construct containing the lux operon under the sigB or sigD promoter and NAT cassette (Figure 1A,B). The *psbA1* gene was selected, as it is practically silent in all conditions used in this study and only expressed in low oxygen conditions [18,19]. Synthetic DNA fragments were purchased from GenScript Biotech (New Jersey, NJ, USA). Then plasmids pP_sigB_lux and pP_sigD_lux were transformed to the glucose tolerant control strain of *Synechocystis* sp. PCC6803. Colonies appeared after one week on BG-11 plates supplemented with nourseothricin (10 μg/mL), and they were streaked on new selective plates once a week for eight weeks. Thereafter, the segregation of PsigB- and PsigD-lux strains was verified with PCR, as described in [10], using the primers 5′-CCGCTACCACCTGTTTTATTA-3′ and 5′- TCGGCCCAGGTGCTCACG- 3′. Finally, the modified areas of the genome in PsigB- and PsigD-lux strains were sequenced to confirm that they were as planned.

### 2.2. Growth at Standard, High Temperature and High Light Conditions

The cells were grown as 30-mL batch cultures in 100-mL Erlenmyer flasks in BG-11 medium that was buffered with 20 mM Hepes, pH 7.5. For standard growth conditions, cells were grown at 32 °C under continuous illumination at the photosynthetic photon flux density (PPFD) of 40 μmol m^−2^s^−1^ in ambient air. The light source was a mixture of fluorescent tubes, light colors 865 and 840 (Osram (Munich, Germany)/Philips (Amsterdam, The Netherlands)). The flasks were shaken at 90 rpm.

To induce long-term, high temperature stress, the OD_730_ of the cell culture was set to 0.06, and 30-mL cell cultures in 100-mL Erlenmyer flasks were grown in Hepes-buffered (pH 7.5) BG-11 medium at 42 °C or at 40 °C, as indicated, PPFD 40 μmol m^−2^s^−1^, ambient air. Cells were grown in Climatic Chamber KK1200 (Pol-Eko Aparatura^®^, Wodzisław Śląski, Poland), the same light source as in standard conditions was used, and the temperature was monitored during experiments. Three independent biological replicates were tested.

To test growth in high light, the OD_730_ of the cell culture was set to 0.06, and 30-mL cell cultures in 100-mL Erlenmyer flasks were grown in Hepes-buffered (pH 7.5) BG-11 medium, PPFD of 750, 500, or 120 μmol m^−2^s^−1^ at 32 °C in ambient air. Cells were grown in a Novotron^®^ TR-225 chamber (Infors AG, Bottmingen, Switzerland), and illuminated using Heliospectra L4A lamp (Heliospectra, Göteborg, Sweden). The lamp setting were 400 nm = 200, 420 nm = 400, 450 nm = 450, 530 nm = 1000, 630 nm = 1000, 660 nm = 900, 735 nm = 0. Neutral density filters 0.3 ND and 0.6 ND (LEE Filters, Hampshire, UK) were used to adjust light intensity. Temperature was monitored during experiments. Three independent biological replicates were tested.

Growth was monitored by measuring OD_730_ once a day. Dense cultures were diluted, so that the measured OD_730_ did not exceed 0.4, and the dilutions were taken into account when the results were calculated. In vivo absorption spectra were measured from 2-day old cells, as described in [16].

### 2.3. Imaging Cell Cultures

Images of cultures were taken with a Canon EOS 250D camera (Canon, Tokio, Japan) equipped with a Canon Compact-Macro EF 50 mm lens (Canon, Tokio, Japan), after 2 and 3 days of growth. For single cell imaging, cells were diluted to OD_730_ of 0.0175, 100 µL of each culture was transferred to a 96-well plate, and images were taken with a Nikon Eclipse Ti2-E-microscope (Nikon, Tokio, Japan) and Nikon DS-Fi3-camera (Nikon, Tokio, Japan).

### 2.4. Activities of sigB and sigD Promoters

The lux reporter gene operon was expressed under the *sigB* or *sigD* promoters. The construction of these strains is described above. Bacterial strains containing the whole lux operon are expected to produce light without the addition of a substrate. However, in *Synechocystis*, we found that the addition of the substrate decanal greatly enhanced the light signal. The CS, PsigB-lux, and PsigD-lux cultures were grown in standard growth conditions for three days. Prior to measurements, 200 μL of cell cultures were supplemented with 0.1 mM decanal in microplate wells (black, flat, clear bottom polystyrol, Corning), after which cells were incubated in standard, high temperature (40 °C) or high light (PPFD 380 μmol m^−2^s^−1^) conditions for 15 min, and then luminescence and OD_730_ were measured with a plate reader (Infinite Pro200, Tecan, Männedorf Switzerland). Luminescence values were calculated relative to OD_730_.

### 2.5. RNAseq Analysis

Thirty-ml cell cultures were grown for three days in standard conditions (to OD_730_ 0.6). Cells were then collected from the standard conditions or treated for 1 h at the PPFD of 750 μmol photons m^−2^ s^−1^ or 1 h at 42 °C, as indicated, maintaining the other conditions same as in the standard conditions. Three biological replicates were prepared for each treatment. A total of 15 mL of cell suspension was poured to a pre-frozen centrifugation tube, and a cell pellet was collected by centrifugation at 7000× *g* for 5 min at 4 °C. Cell pellets were frozen in liquid nitrogen. Isolation of total RNA was done with the hot phenol method [20]. Sequencing libraries for total RNA samples were prepared using Illumina TruSeq Stranded mRNA kit protocol. The quality of the library was confirmed using the Advanced Analytical Fragment Analyzer (Advanced Analytical Technologies, Heidelberg, Germany), and the concentrations of the libraries were quantified with Qubit^®^ Fluorometric Quantitation (Life Technologies, ThermoFisher, Waltham, MA, USA). Sequencing was done with a HiSeq2500 Next Generation Sequencing platform at the Finnish Functional Genomics Centre. The quality control of raw sequencing reads was performed with FastQC (http://www.bioinformatics.babraham.ac.uk/projects/fastqc/ accesed on 20 November 2020). Reads were aligned to the *Synechocystis* sp. PCC 6803 (Moscow strain) reference genome (Ensemble release 35) using HISAT2 [21]. HTseq-count [22] was used to calculate summarized read counts for each gene, except that the two ribosomal RNA operons were excluded from the analysis. DESeq2 [23] was used to identify differentially expressed genes. Wald test statistic is used for hypothesis testing in DESeq2. Genes with a false discovery rate, <0.05, were considered to show significantly changed expression. Data analysis was done with the CSC’s Chipster v.4 platform [24]. We considered the gene to be up- or down-regulated in a mutant strain if its expression was at least two-fold up-regulated (log_2_ fold change ≥ 1) or down-regulated to one half or less (log_2_ fold change ≤ −1), compared to the CS strain in the same conditions, respectively.

For regulon analysis, we chose genes showing a log_2_ fold change ≥ 1 or ≤ −1 in at least one of the mutant strains, compared to the control strain in the same conditions. Genes were assigned to the SigB regulon if they were significantly down-regulated both in ΔsigB and ΔsigBCE strains, but not in the other strains (up-regulation in ΔsigCDE was allowed), or significantly up-regulated both in ΔsigB and ΔsigBCE strains, but not in the other mutant strains (down-regulation in ΔsigCDE was allowed). Genes were assigned to the SigD regulon if they significantly down-regulated both in ΔsigD and ΔsigCDE strains, but not in the other mutant strains (up-regulation in ΔsigBCE was allowed), or significantly up-regulated both in ΔsigD and ΔsigCDE strains, but not in the other mutant strains (down-regulation in ΔsigBCE was allowed). Genes simultaneously significantly down- or up-regulated in both triple mutant strains, but not in the single mutants, were considered to belong the SigC and/or SigE regulons. Since the ΔsigC and ΔsigE strains were not included in the study, we cannot tell whether a gene belongs to the SigC or the SigE regulon. To visualize the results, heat maps were created using a web tool [25]. RNAseq data are available in GEO (accession GSE192357).

## 3. Results

### 3.1. Response of Promoters of sigB and sigD Genes to Heat and High Light

To study the specific roles of SigB and SigD factors, we first measured the response of *sigB* and *sigD* promoters to heat and high light. To that end, the predicted promoter regions of *sigB* and *sigD* genes were cloned in front of the *luxCDABE* operon from *Photorhabdus luminescens*, and the non-essential *psbA1* gene was replaced from *Synechocystis* genome with these constructs to produce PsigB- and PsigD-lux strains (Figure 1A–C). The whole *luxCDABE* operon, as such, is able to induce production of luminescence in *E. coli*. However, in *Synechocystis*, measured luminescence levels were low, without the addition of the substrate decanal; the decanal was, therefore, added to cell samples 15 min prior to measurements.

The background luminescence signal from the control strain without the *lux* construct was negligibly low in all studied conditions (Figure 1D). Luminescence levels remained low in PsigB- and PsigD-lux strains in standard growth conditions (Figure 1D). In high light, luminescence of the PsigD-lux strain increased almost five-fold, whereas that of the PsigB-lux strain was tripled (Figure 1D). At high temperature, the luminescence levels of PsigB- and PsigD-lux strains were 2.2- and 1.4-fold higher than in the standard growth conditions, respectively. These results indicate that both *sigB* and *sigD* genes are activated by high light and temperature, suggesting that SigB and SigD play roles in acclimation to these stress conditions.

### 3.2. Comparison of Transcript Profiles of sigB and sigD Deletion Strains in Standard Growth Conditions

To better understand the roles of SigB and SigD σ factors, we compared transcriptomes of ΔsigB, ΔsigD, ΔsigBCE (with SigD as the only functional group 2 σ factor), and ΔsigCDE (SigB as the only functional group 2 σ factor) strains. Total RNA was analyzed from three biological replicates with RNAseq. In addition to our standard growth conditions, we also analyzed samples after 1 h treatments at high temperature (42 °C) or light (PPFD 750 μmol m^−2^s^−1^). We selected 1 h treatment to allow the accumulation of SigB and SigD proteins after the stress-induced induction of *sigB* and *sigD* genes [5].

**Figure 2:**
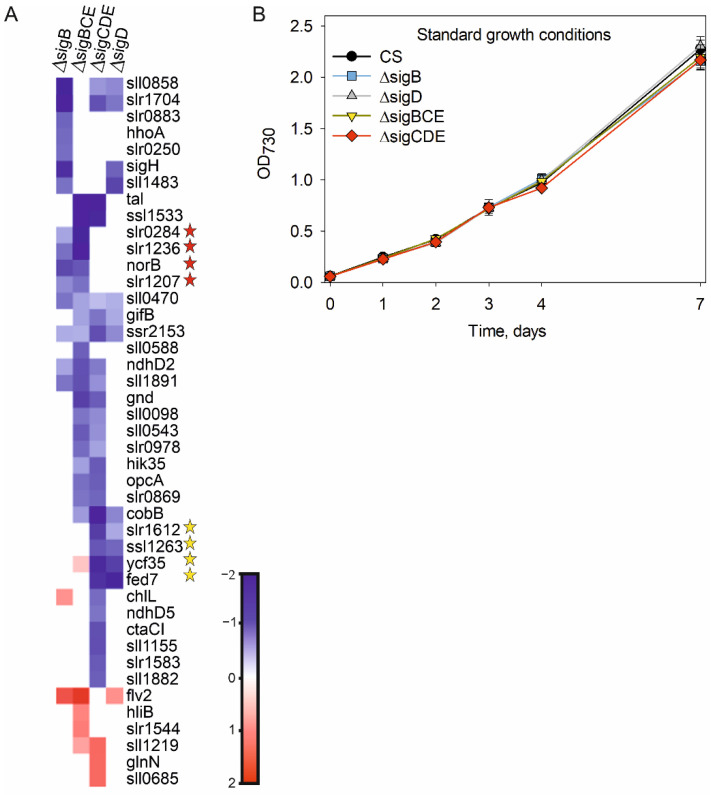
Gene expression changes and growth of ΔsigB, ΔsigD, ΔsigBCE, and ΔsigCDE strains in standard growth conditions. (**A**) A heat map showing genes whose transcripts were at least two-fold more abundant or reduced to half or less in at least one of the mutant strains (ΔsigB, ΔsigD, ΔsigBCE, and ΔsigCDE), compared to the control strain of *Synechocystis* sp. PCC6803 in the standard growth conditions. The bar shows color codes for log_2_ values of the transcript fold changes. Genes belonging to the SigB regulon (red stars) or the SigD regulon (yellow stars) are indicated. (**B**) Growth of the glucose tolerant control strain (CS) of *Synechocystis* sp. PCC 6803, and ΔsigB, ΔsigD, ΔsigBCE, ΔsigCDE mutant strains in standard growth conditions, defined as: constant light PPFD 40 μmol m^−2^s^−1^, 32 °C, ambient air, BG-11 medium buffered with 20 mM Hepes-NaOH, pH 7.5. Three independent biological replicates were measured (**A**,**B**) and the error bars denote SE (**B**).

All genes showing statistically significant difference in at least one of the mutant strains, compared to the CS strain in standard growth conditions, are listed in Supplemental Table S1. For further analysis, we selected only genes whose expression was at least twice of that measured in CS or down-regulated to only half of that measured in CS in at least one of the mutant strains. Using these criteria, 43 genes showed differential expression in at least one of the mutant strains, compared to the control strain (Supplemental Table S1; Figure 2A); the inactivated group 2 σ factors were not included in these analyses. In the triple mutants, more genes (18 in ΔsigBCE and 22 in ΔsigCDE) showed at least 2-fold differential expression than in the single mutants (12 in ΔsigB and 5 in ΔsigD). The majority of differentially expressed genes, 24, belongs to Cyanobase categories unknown or hypothetical.

We selected genes showing either two-fold up- or down-regulation in at least one of the mutant strains for regulon analysis. The criteria placing genes to different regulons are described in Section 2.5. Note that when counting differently expressed genes in a mutant strain, we only count those ones that show at least two-fold expression differences, whereas in regulon analysis genes whose expression difference is less than two-fold, but significant, were counted. In standard conditions, the SigB regulon comprises of the *norB*, *slr0284*, *slr1236*, and *slr1207* genes, whose expression was down-regulated in ΔsigB and ΔsigBCE (Figure 2A). SigD-dependent transcripts were the *slr1612*, *ssl1263*, *fed7*, and *ycf35* genes (Figure 2A). Nine genes (*tal*, *ssl1533*, *gnd*, *sll0098*, *sll0543*, *slr0978*, *hik35*, *opcA*, and *slr0869*) were down-regulated and one (*sll1219*) was up-regulated in both triple inactivation strains, but not in the single mutants, suggesting that these genes are controlled by SigC or SigE factors rather than by SigB or SigD factors (Figure 2A). Two of the genes, *sll0407* and *ssr2153,* were significantly down-regulated in all mutant strains. Overall, transcriptional differences between the strains were small; accordingly, similar growth rates were measured for all strains in standard growth conditions (Figure 2B).

### 3.3. SigB Regulon Is Bigger Than SigD Regulon at High Temperature

**Figure 3:**
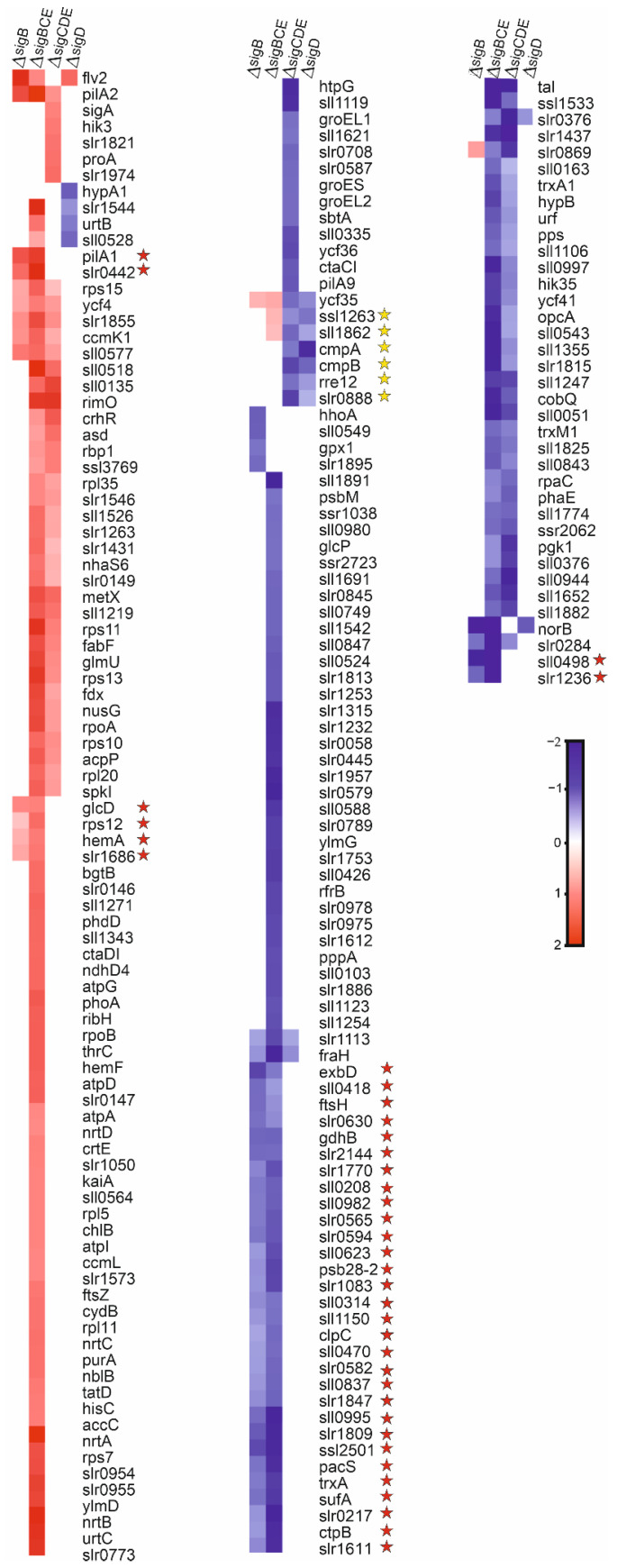
A heat map showing genes whose transcripts were at least two-fold up- or down- regulated in at least one of the mutant strains, in comparison to the CS strain at high temperature. Cells were treated at 42 °C for 1h, in otherwise standard conditions. The bar shows color codes for log_2_ values of the transcript fold changes. Genes belonging to the SigB regulon (red stars) and the SigD regulon (yellow stars) are indicated.

In a high temperature treatment, the number of differently expressed genes in the mutant strains was higher than in standard growth conditions (Supplemental Table S2). In total, 221 genes were at least two-fold up- or down-regulated in at least one of the mutant strains, compared to the control strain after 1 h treatment at 42 °C (Figure 3). In the ΔsigB strain 25 genes and in ΔsigD strain 7 genes were differently regulated. In ΔsigCDE and ΔsigBCE, 51 and 172 genes were differently expressed, in comparison to the CS strain, respectively. Of these genes, 27 genes were up-regulated and 31 down-regulated in both triple inactivation strains but showed normal expression in both single mutants (Figure 3), indicating that the expression of 58 (27 + 31) genes was dependent on SigC or SigE factors, not SigB or SigD factors.

**Figure 4:**
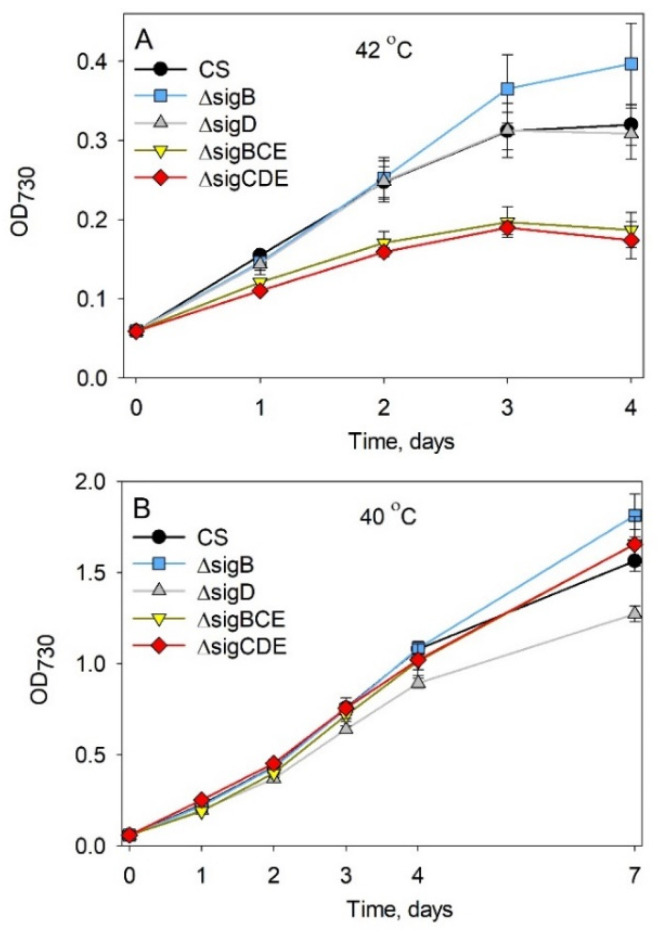
Growth of *Synechocystis* CS, ΔsigB, ΔsigD, ΔsigBCE, and ΔsigCDE strains at high temperature. The temperature was 42 °C (**A**) or 40 °C (**B**), and the other conditions were the same as in the standard conditions. At least three independent biological replicates were measured, and the error bars denote SE.

Only a few genes can be considered to belong to the SigD regulon at high temperature. The SigD regulon includes the down-regulated genes *ycf35, rre12*, *ssl1263*, *sll1862*, and *slr0888*, as well as the *cmp* operon (Figure 4). The *cmp* genes encode the bicarbonate transporter BCT1, and Rre12 has been connected to chemotaxis in unfavorable conditions, whereas the biological functions of other genes in the regulon remains to be solved.

The SigB high-temperature regulon comprised of 36 genes. The up-regulated genes include *pilA1* (a major structural protein of the pilus), *glcD* (a subunit of glycolate oxidase)*, rps12* (a protein of the small ribosomal subunit), *hemA* (transfer RNA-Gln reductase), and the *slr0442* and *slr1686* genes. The down-regulated genes include *exbD* (a putative biopolymer export protein), *ftsH* (a PSII repair machinery metalloprotease), *gdhB* (a putative glucose dehydrogenase), *psb28-2* (Photosystem II subunit), *clpC* (ATPase subunit of Clp protease), *pacS* (copper-transporting P-type ATPase), *trxA* (thioredoxin), *sufA* (FeS cluster assembly accessory/regulatory protein), and *ctpB* (periplasmic carboxyterminal protease) genes, as well as 23 open reading frames encoding proteins with unknown functions (Figure 3). As so many genes of this regulon belong to categories hypothetical/unknown, it remains to be elucidated how the SigB regulon actually helps cells to acclimate to high temperature. Gene expression results suggest that the SigB factor might play a more important role at high temperature than the SigD factor, as the SigB regulon is six times bigger than the SigD regulon. However, it should be noted that more genes actually belong to the SigC and SigE regulons (58 genes) than the SigB and SigD regulons (42 genes) at high temperature.

The importance of SigB and SigD during long-term exposure to high temperature was tested by growing cells at 42 °C. In comparison to the standard growth conditions, growth of all strains slowed down at 42 °C. After the first day at 42 °C, the OD_730_ was only half of that measured in the standard growth conditions (Figure 4A). Both triple inactivation strains grew more slowly than the CS strain at 42 °C, whereas ΔsigD and ΔsigB strains grew like the CS strain. The growth rates of all strains decreased upon prolonged high temperature treatment; after the third day, the cultures started to decline and died in less than one week. These results indicate that 42 °C is a too high temperature for maintaining *Synechocystis* cells. At 40 °C, CS cells continued to grow for a whole one-week experiment, indicating that 40 °C is a permissive condition (Figure 4B). At 40 °C, growth of the triple inactivation strains did not differ from that measured for CS. Single mutants grew like the CS for the first three days; after that, the growth of the ΔsigD strain decelerated more than that of the other strains.

### 3.4. The SigD Regulon Is Bigger in High Light Than the SigB Regulon

**Figure 5:**
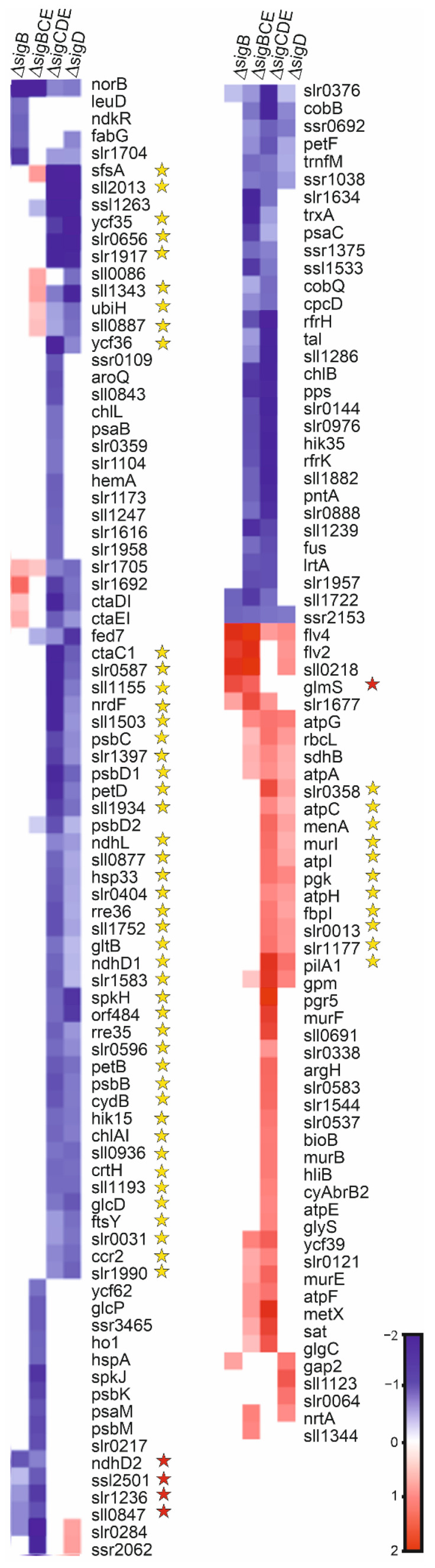
A heat map showing genes whose transcripts were two-fold or more up- or down-regulated, at least in one of the mutant strains ΔsigB, ΔsigD, ΔsigBCE, or ΔsigCDE, in comparison to the CS strain in high light. Cells were treated with PPFD 750 μmol m^−2^s^−1^at 32 °C for 1h in otherwise standard conditions. The bar shows color codes for log_2_ values of the transcript fold changes. Genes belonging to the SigB (red stars) or the SigD regulons (yellow stars) are indicated.

**Figure 6:**
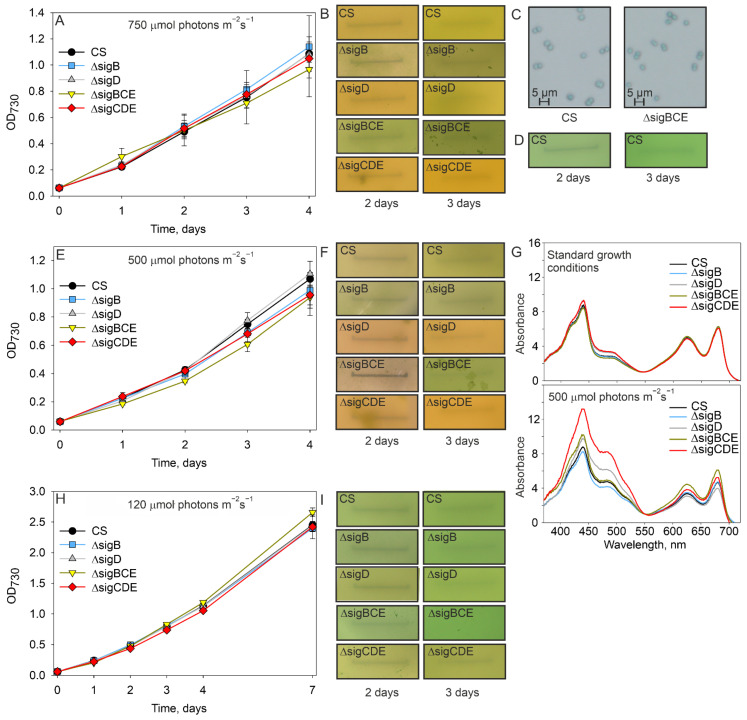
Growth of *Synechocystis* CS, ΔsigB, ΔsigD, ΔsigBCE, and ΔsigCDE strains in high light. The growth curves were measured at the PPFD of 750 (**A**), 500 (**E**), and 120 (**H**) μmol m^−2^s^−1^ and cultures were photographed on days 2 and 3 (**B**,**F**,**I**). Cells grown in high light were resuspended carefully and photographed under microscope (**C**). The CS strain was photographed in standard growth conditions on days 2 and 3 (**D**). The in vivo absorption spectra (**G**) were measured from two-day old cells, grown in standard growth conditions or high light (PPFD 500 μmol photons m^−2^s^−1^). Three independent biological replicates of growth curves were measured, and the error bars denote SE. The line in the photographs is 1 cm.

After the high light treatment (1h, PPFD 750 μmol m^−2^s^−1^), 8 genes were at least two-fold down-regulated and 5 genes were two-fold up-regulated in ΔsigB, compared to the CS strains, whereas, in ΔsigD, 29 genes were down-regulated and 9 were up-regulated (Supplemental Table S3, Figure 5). In ΔsigBCE and ΔsigCDE strains, 38 and 80 genes were down-regulated, respectively, whereas only 9 and 34 genes were up-regulated in those strains, respectively. The SigC or SigE regulons (regulated in both triple inactivation strains, but not in single mutants) comprise of 23 down-regulated and 7 up-regulated genes (Figure 5), 1 and 6 of which were also, respectively, down- or up-regulated at high temperature.

The SigB high-light regulon comprised of an up-regulated *glm* gene (glucose-1-phosphate adenylyltransferase) and down-regulated *ndhD2*, *ssl2501*, *slr1236*, *sll0847,* and *slr0284* genes (Figure 5). For these, only *ssl2501* belongs to the SigB high-temperature regulon.

The SigD high light regulon included 51 down-regulated and 11 up-regulated genes (Figure 5). Up-regulated SigD regulon genes comprised of the *pilA1*, *atpC, atpI, atpH*, *menA* (plastoquinone synthesis)*, murI* (peptidoglycan synthesis)*, pgk* (phosphoglycerate kinase)*, fbpI* (Lys propinyilation), *slr0358, slr0013,* and *slr1177* genes. Interestingly, the *pilA1* gene was found among the SigB regulon genes at high temperature. In addition, many ATP synthase genes were up-regulated in high light in the ΔsigD and ΔsigCDE strains missing the SigD factor, whereas, at high temperature, ATP synthase genes were up-regulated in the ΔsigBCE strains that contain SigD as only the functional group 2 σ factor. These results indicate that the expression of a particular gene might be dependent on different σ factors, depending on prevailing environmental conditions.

SigD regulon in high-light included four genes that were down-regulated in ΔsigD and ΔsigCDE and up-regulated in ΔsigBCE: a ubiquinone synthesis gene *ubiH*, a sugar catabolism gene *sfsA*, and two genes (*sll0877* and *sll1343*) with unknown function. Many photosynthesis- and respiration-related genes were down-regulated in high light in strains missing SigD; these genes included *psbB, psbC, psbD1* and *psbD2* (Photosystem II), *petB* and *petD* (cytochrome b_6_f complex), *ndhD1* and *ndhL* (NADH dehydrogenase), *chlAI* and *crtH* (pigment synthesis), and *ctaC1* (respiration) (Figure 5). In addition, the down-regulated were *hsp33* (chaperone), *ccr2* (cold stress tolerance gene), *rre35, rre36* and *hik15* (two component response regulator proteins), *spkH* (a ser/thr kinase), *ftsY* (a protein translocation/insertion protein), *gltB* (a NADH-dependent glutamate synthase), *glcD* (a glycolate oxidase), *nrdF* (synthesis of purine ribonucleotides), *ycf35*, and 17 genes characterized as unknown or hypothetical (Figure 5).

We next tested the growth of mutant strains at the same high light intensity, where RNAseq analysis was carried out. Growth of cells at the PPFD of 750 µmol photons m^−2^s^−1^ (Figure 6A) induced the formation of cell aggregates in all strains and, especially, strains ΔsigB and ΔsigBCE produced numerous aggregates (Figure 6B). However, mixing cell cultures for a couple of minutes by pipetting broke up the aggregates (Figure 6C), allowing for OD_730_ measurements.

In standard conditions, *Synechocystis* cultures have an intense green color (Figure 6D), but in high light colors of cultures turn to different tones of olive and yellowish-brown, depending on the mutant strain (Figure 6B). Although cell cultures had different appearances, the growth of the different strains did not differ much, as only ΔsigBCE grew slightly faster than the other strains on the first day (Figure 6A). As a high light treatment at PPFD 750 µmol photons m^−2^s^−1^ did not improve growth, compared to the standard conditions, we next tested if a reduction of irradiation to PPFD 500 µmol photons m^−2^s^−1^ would improve growth.

According to the OD_730_ measurements, the growth rate at PPFD 500 µmol photons m^−2^s^−1^ remained similar as at 750 µmol photons m^−2^s^−1^, and a great variability in culture colors between the strains was observed (Figure 6E,F). Obviously, high light-induced pigment changes differ between the mutant strains. In vivo absorption spectra measurements showed that contents of phycobilin and chlorophyll a were similar in all strains in standard growth conditions, but carotenoid content was higher in ΔsigCDE and ΔsigD strains than in the other strains (Figure 6G). The olive color of the CS strain in high light was due to simultaneous increase of carotenoid content and reduction of chlorophyll a and phycobilin contents, compared to standard growth conditions (Figure 6G). The intense yellowish-brown color of the ΔsigCDE strain in high light is due to high accumulation of carotenoids and less pronounced reduction of chlorophyll a and phycobilin than in the CS strain (Figure 6G). The ΔsigD strain shows a yellowish appearance in high light (Figure 6B,F). The absorption spectra show that carotenoids are highly induced in the ΔsigD strain in high light, whereas chlorophyll a and phycobilin are more drastically reduced than in the other strains; together, these pigment changes explain the yellowish color of the ΔsigD culture in high light. Interestingly, the behavior of the ΔsigBCE strain that contains SigD as the only functional group 2 σ factor was opposite to that of ΔsigD strain. The ΔsigBCE strain remained greener in high light than the other strains (Figure 6B,F), because it retained more chlorophyll a and phycobilin that the control strain (Figure 6G). These results suggest that SigD plays an important role in the adjustment of photosynthetic pigments, according to light intensity.

Finally, we reduced PPFD to 120 µmol m^−2^s^−1^. At PPFD 120 µmol m^−2^s^−1^, the CS strain grew 15% faster than in the standard growth conditions (Figure 6H and Figure 2B). The ΔsigBCE strain grew slightly better than the other strains, but otherwise all mutants grew like the CS. Color differences between the strains were not as notable as in higher light conditions, but ΔsigCDE and ΔsigD strains remained more yellowish than the other strains (Figure 6H).

## 4. Discussion

The construction of robust cyanobacteria strains is one of the current challenges for biotechnology applications. Inactivation of the three other group 2 σ factors leads to two-fold overproduction of the remaining SigB or SigD factor in the ΔsigCDE and ΔsigBCE strains, respectively [16]. As a previous study [16] has shown that ΔsigCDE and ΔsigBCE strains tolerate chemically-induced oxidative stress better than the CS strain, we considered the possibility that extra SigB or SigD factors in the triple inactivation strain would protect cells in high light or temperature conditions. However, that was not the case; actually, our results point to the involvement of all group 2 σ factors in different stress conditions. To test if the fitness of the cells in some stress conditions could be improved by overexpressing a particular σ factor should, therefore, be done with a wild-type strains as the host strain.

**Figure 7:**
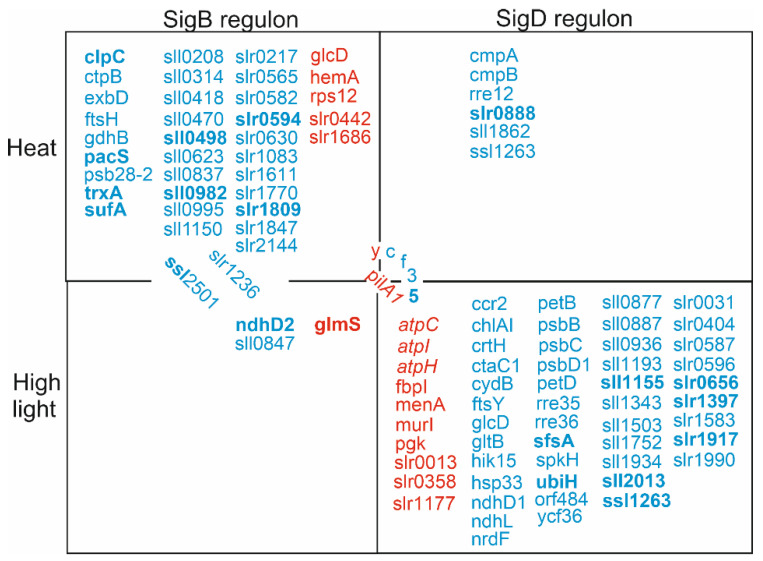
SigB and SigD regulons at high temperature (42 °C) and light (PPFD 500 μmol photons m^−2^s^−1^). Genes shown in blue were down-regulated, and those in red up-regulated. Genes with bold font were up-regulated when the transcriptome of the CS strain was compared in high light or temperature to that of the CS strain in the standard growth conditions, and italics indicate genes down-regulated in the CS strain in the test conditions.

The comparison of transcriptomes of ΔsigB, ΔsigD, ΔsigCDE, and ΔsigBCE strains, in standard conditions and after 1 h of treatment in high light (750 μmol photons m^−2^s^−1^) or at temperature (42 °C), revealed new features and confirmed old findings of gene regulation in *Synechocystis*. Group 2 σ factors indeed play only marginal roles in standard growth conditions but are important for acclimation to suboptimal conditions [6]. Our results revealed that although SigB and SigD factors are close homologs [8], their regulons do not overlap (Figure 3, Figure 5 and Figure 7). Furthermore, the comparison of up- and down-regulated genes after high light and temperature treatments showed that both SigB and SigD regulate different sets of genes in high light and temperature (Figure 7). Although our study did not focus on SigC or SigE regulons, transcriptomes of triple mutants contained numerous sugar catabolism and other genes belonging to SigE regulon [26,27], and our results indicate that the SigE regulon does not show similar high variation between the studied environmental condition as SigB and SigD regulons. As both triple inactivation strains grew slowly at high temperature, our results suggest that the SigE-dependent regulation of sugar catabolic genes and energy production by respiration might play an important role in high temperature stress, when temperature-sensitive photosynthesis [28] might not be fully functional. A detailed analysis of the photosynthetic and respiration reactions in heat stressed σ factor mutant strains would be interesting.

Both triple inactivation strains grew more slowly than the CS or single inactivation strains at 42 °C (Figure 4A), but this disadvantage of triple mutants disappeared at 40 °C (Figure 4B). In triple mutants, this growth behavior might mainly be due to the missing SigC factor, as the ΔsigC strain has been shown to have serious growth defects at 43 °C, but not at 38 °C [28]. SigC is a growth restricting σ factor [29,30,31], and the formation of RNAP-SigC holoenzymes at high temperature occurs more regularly than at optimal temperatures [5]. In accordance with enhanced formation of growth restricting RNAP-SigC holoenzymes, the growth rate of the CS strain is very slow at 42 °C, in comparison to the optimal growth temperature (Figure 2 and Figure 4A). RNAP-SigC induced adjustments of gene expression are important for heat acclimation. At high temperatures, numerous household genes, including transcription, translation, pigment synthesis, and photosynthesis genes, are not normally down-regulated upon temperature upshift in triple inactivation strains, and cells show early growth cessation at high temperature (Figure 4). Obviously, the SigC controlled growth restriction is a central process for high temperature acclimation and retaining a functional SigC is a prerequisite for engineering temperature robustness of *Synechocystis* strains.

The amount of the RNAP-SigB holoenzyme is highly up-regulated at high temperature, whereas the amount of RNAP-SigD remains constant [5]; accordingly, more genes belong to the SigB regulon than to the SigD regulon at high temperature (Figure 3 and Figure 7). The ΔsigB strain shows a low survival rate after a short term lethal temperature treatment (48 °C) and defects in the acquisition of acquired thermotolerance [10], but growth differences were not detected in milder heat stress (Figure 4; [10,11]). The overproduction of SigB in wild-type backgrounds has shown to increase heat resistance of *Synechocystis* cells [32], but we do not see the same effect in the ΔsigCDE strain that contains an abnormally high amount of SigB [16], but is missing the other group 2 σ factors.

Our results point to high flexibility and variability of high light acclimation. The growth rate of *Synechocystis* cells, at standard growth conditions (PPFD 40 μmol photons m^−2^s^−1^) and in all tested high light conditions (120, 500 and 750 μmol photons m^−2^s^−1^), remained similar in all mutant strains. One consequence of high light treatment is an accelerated rate of PSII damage. However, all our strains contained either the SigB or SigD factor, and it has been previously shown that the presence of either SigB or SigD can guarantee the normal high light-specific up-regulation of *psbA* genes that is required for efficient repair of photodamaged photosystem II centers [15].

The *sigD* gene is induced in high light [9,14], and the RNAP-SigD holoenzyme is accumulated in high quantities in high light [5], which explains why the SigD regulon is big in high light (Figure 5 and Figure 7). However, cells without the SigD factor did not show clear growth rate reductions in high light (Figure 6). The ΔsigBCE strain shows up-regulation of RNAP-SigD holoenzyme formation, compared to the CS strain (Hakkila et al., 2019), and ΔsigBCE cells grow faster in the beginning of high light treatment, but this growth advantage disappeared after long high light treatment (Figure 6A). The ΔsigBCE strain remains greener in high light than the other strains because the phycobilins and chlorophyll a are not degraded as much as in the other strains and the carotenoid content remains low. Previous studies have shown that the ΔsigBCE strain is more resistant against chemically-induced singlet oxygen and H_2_O_2_ stresses than the control strain [3,16]. We have previously shown that the amount of the SigD protein is two times as high in the ΔsigBCE strain as the CS strain [16], suggesting that overproduction of SigD might offer protection against some reactive oxygen species. Obviously, the extra protection in the ΔsigBCE strain is not due to carotenoids, as the carotenoid content is lower in ΔsigBCE strain than in the other strains in high light (Figure 6G). A high tendency of ΔsigBCE strain to form cell aggregates might function as a strategy to escape too high light. Both strains without the SigD factor (ΔsigD and ΔsigCDE) were characterized by high accumulation of carotenoids (Figure 6G). Carotenoids are especially important protective pigments, and *Synechocystis* cells are not viable in light without carotenoids [33]. Hence, the accumulation of carotenoids could be seen as another strategy to survive in high light.

The *sigB* gene is induced in high light (Figure 1), although to a lesser extent than the sigD gene, and similar induction of the RNAP-SigB holoezyme is not seen in high light as is seen for RNAP-SigD [3,16]. Our previous results have shown that high amounts of carotenoids, as well as Flv2, Flv4, and Sll0218 proteins, in ΔsigCDE reduce the production of singlet oxygen and confer resistance of PSII against light-induce damage; simultaneously, reduced contents of glutathione and Flv1/3 proteins led to more pronounced oxidative damage in ΔsigCDE than in the control strain. Due to these opposing effects, the ΔsigCDE grew like a control strain in high light but showed superior growth in H_2_O_2_ stress [16]. Our results show that, actually, triple inactivation strains containing only SigD or SigB managed quite well in high light because their acclimation to some features of high light stress was superior, compared to the CS strains, and compensated for problems to acclimate other features of high light stress.

## 5. Conclusions

Our results show that the SigB and SigD factors control unique regulons with negligible overlay. Furthermore, the SigB regulon was found to be seven times bigger in heat stress than in high light and mainly comprised of genes with unknown functions. SigD regulon was bigger in high light than in heat stress and contained a different set of genes in different stress conditions. Our results point to the flexibility of the high light acclimation mechanism of *Synechocystis* and indicate that the SigD factor plays a role in adjusting pigment content, according to prevailing light conditions.

## Data Availability

RNAseq data are available in GEO, accession GSE19235. https://www.ncbi.nlm.nih.gov/geo/query/acc.cgi?acc=GSE192357 (accessed on 20 January 2021).

## Supplementary Materials

The following supporting information can be downloaded at: https://www.mdpi.com/article/10.3390/life12020162/s1, Table S1: A list of genes whose expression is different (*p* < 0.05) at least in one of the mutant strains compared to the control strain (CS) in standard growth conditions., Table S2: A list of genes whose expression in at least in one mutant shows differential expression compared to that in the control strain after 1 h treatment at 40°C., Table S3: A list of genes whose expression is significantly different (*p* < 0.05) compared to the control strain in at least of the mutant strains after 1 h treatment at photosynthetic photon flux density of 750 mmol m^−2^s^−1^.
